# Both the COMT Val158Met single-nucleotide polymorphism and sex-dependent differences influence response inhibition

**DOI:** 10.3389/fnbeh.2015.00127

**Published:** 2015-05-19

**Authors:** Valentina Mione, Sonia Canterini, Emiliano Brunamonti, Pierpaolo Pani, Federica Donno, Maria Teresa Fiorenza, Stefano Ferraina

**Affiliations:** ^1^Department of Physiology and Pharmacology, Sapienza UniversityRome, Italy; ^2^Department of Psychology, Section of Neuroscience, Sapienza UniversityRome, Italy; ^3^“Daniel Bovet” Neurobiology Research Center, Sapienza UniversityRome, Italy

**Keywords:** inhibition, proactive control, motor, sex differences, COMT

## Abstract

Reactive and proactive controls of actions are cognitive abilities that allow one to deal with a continuously changing environment by adjusting already programmed actions. They also set forthcoming actions by evaluating the outcome of the previous ones. Earlier studies highlighted sex-related differences in the strategies and in the pattern of brain activation during cognitive tasks involving reactive and proactive control. To further identify sex-dependent characteristics in the cognitive control of actions, in this study, we have assessed whether/how differences in performance are modulated by the COMT Val158Met single-nucleotide polymorphism (SNP), a genetic factor known to influence the functionality of the dopaminergic system—in particular, at the level of the prefrontal cortex. Two groups of male and female participants were sorted according to their genotype (Val/Val, Val/Met, and Met/Met) and tested in a stop signal task, a consolidated tool for measuring executive control in experimental and clinical settings. In each group of participants, we estimated both a measure of the capacity to react to unexpected events and the ability to monitor their performance. The between-group comparison of these measures indicated a poorer ability of male individuals and Val/Val subjects in error-monitoring. These observations suggest that sex differences in inhibitory control could be influenced by the efficiency of COMT and that other sex-specific factors have to be considered. Understanding the inter-group variability of behavioral and physiological correlates of cognitive control could provide more accurate diagnostic tools for predicting the incidence and/or the development of pathologies, like ADHD, or deviant behaviors, such as drug or alcohol abuse.

## Introduction

Inhibitory control is an important component of executive functions. In both humans and non-human primates, a network of cortical-subcortical brain areas, with key nodes in the frontal lobe, basal-ganglia, and cerebellum, sustains this important neurocognitive function (Rieger et al., 2003; Chambers et al., 2009; Schall and Godlove, 2012; Battaglia-Mayer et al., 2014; Brunamonti et al., 2014; Stuphorn, 2015). However, studies in humans using imaging approaches have provided evidence of considerable variability among individuals in the activation of this network (Garavan et al., 2006; Li et al., 2006, 2009; White et al., 2014). Such variability has been found to be associated with both personality traits and the risk of developing psychopathologies, pinpointing its clinical relevance to the control of response inhibition (for a review, Robbins et al., 2012). For example, studies in humans have highlighted a disparate pattern of neural activation between males and females during the execution of tasks exploiting inhibitory control of actions, such as the countermanding task (Garavan et al., 2002; Aron and Poldrack, 2006; Li et al., 2006, 2009; White et al., 2014), suggesting the existence of sex-dependent differences in neuroanatomical and neurobiological substrates of inhibitory control. This possibility is supported by evidence that males are more prone to experience disorders of impulse control, such as attention deficit hyperactivity disorder (ADHD), substance abuse, and conduct disorders, compared to females (Newman et al., 2005; Eme, 2007; Kessler et al., 2007; White et al., 2014). In spite of the relevance to a better understanding of these disorders, the issue of sex-dependent variability in the control of inhibitory responses has been poorly investigated so far (Thakkar et al., 2014).

Dysregulation in the fronto-striatal dopaminergic system is recognized as central in many of the disorders that display defective inhibitory control (Rubia et al., 2011; Hart et al., 2013). Genetic variations of the dopaminergic system have been proposed to contribute to the manifestation of these disorders. For example, the well-established cross-talk between cortical and striatal dopaminergic transmission is specifically influenced by a genetic variation in the dopamine-metabolizing enzyme catechol-ortho-methyl-transferase (COMT) at the level of the prefrontal cortex (Tunbridge et al., 2004; Meyer-Lindenberg et al., 2006; Dickinson and Elvevåg, 2009). For instance, among others, the COMT Val158Met single-nucleotide polymorphism (SNP) is known to influence thermal stability and consequently COMT activity (Chen et al., 2004). In the human prefrontal cortex, Met158 homozygotes display lower (35–50% reduction) COMT activity compared to Val158 homozygotes (Chen et al., 2004) that corresponds to increased availability of dopamine in the extracellular space. Coherent with this observation, a slight advantage of subjects carrying the Met allele has been reported in neuropsychological studies evaluating prefrontal cortex-regulated cognitive functions using either the Wisconsin Card Sorting Test (WCST) or the N-Back task (for a recent review, see Dickinson and Elvevåg, 2009).

However, the COMT activity in the prefrontal cortex of human males has been reported to be higher than in females, independent of the Val158Met and other SNPs (Chen et al., 2004). In addition, male COMT knockout mice (COMT^−/−^), but not females, show increased levels of dopamine compared to male COMT^+/+^ and COMT^+/−^ mice (Gogos et al., 1998). This evidence indicates that sex-dependent variability in the control of inhibitory responses needs to take into account both genes involved in dopamine activity and other sex-related factors that could influence either dopamine availability or the inhibitory process. In this respect, a sexually dimorphic influence of COMT Val158Met alleles on psychiatric phenotypes has been described (review in Harrison and Tunbridge, 2008). Indeed, the Met allele has been associated with obsessive-compulsive disorders (OCDs) in males but not in females (Karayiorgou et al., 1999).

Here, we have investigated the relationship between sex and inhibitory control in a group of healthy subjects sorted for COMT variation by determining their behavioral characteristics in a countermanding task (Logan, 1994). The countermanding task allows one to study response inhibition with high behavioral control and to easily assess both reactive and proactive controls (Rabbitt and Phillips, 1967; Verbruggen and Logan, 2009). We hypothesized that sex-dependent differences were likely to emerge for specific aspects of cognitive control—reactive vs. proactive—and that they might be affected differently by the COMT Val158/Met SNP. This possibility is consistent with differences in the anatomical and physiological substrates of reactive and proactive controls, as recently suggested (Aron, 2011; Schall and Godlove, 2012; Pani et al., 2014).

We show that no relationship exists between either the sex of participants or the COMT genotype and the reactive component of response inhibition. Conversely, an important effect of both sex and genotype emerges when proactive control is assessed, further strengthening the hypothesis that different neurobiological substrates control reactive and proactive components of response inhibition.

## Materials and Methods

### Participants and Task

One hundred thirty-two subjects were randomly selected from a cohort of 150 subjects previously genotyped for COMT Val158Met classification as participants of a different study in our lab. Subjects were selected so as to form two groups comparable for sex (male, *n* = 71 and female, *n* = 61) and tested in a countermanding (stop) task. All participants had normal or corrected-to-normal vision, were unrelated, and were Caucasian. None had a history of significant drug or alcohol abuse, head trauma, or significant medical illness in the past 6 months. All subjects signed informed consent, in accordance with the protocol approved by the ethics committee of the Department of Psychology of Sapienza University. Participation in the study was on a voluntary basis without payment or non-monetary reward. One hundred thirty participants (males, *n* = 69 and females, *n* = 61) were included in the final analyses, excluding two male subjects that failed in performing the task as instructed. Demographic details of these subjects are reported in Table 1. Most of them were right-handed, as determined by the Edinburgh Handness Inventory (EHI; Oldfield, 1971); the number of left-handed subjects was similar in the two groups (see Table 1). Genotype frequencies, within male and female groups, were consistent with Hardy-Weinberg equilibrium (males, *χ*^2^ = 0.027; *p* = 0.90; females *χ*^2^ = 0.14; *p* = 0.70). Males were significantly older than females (*t*-test). Although the difference was negligible (about 1 year), we decided to add age as a covariate in all the following analyses (ANCOVA and repeated-measures ANCOVA).

**Table 1:** **Demographic details of experimental participants**.

	Males (*n* = 69)	Females (*n* = 61)	Statistic (*p*)
**Age**	26.88, 2.74	25.64, 2.75	*t* = −2.55
(Mean, SD)			(*p* = 0.01)
**Genotype**	18; 35; 16	14; 29; 18	*χ*^2^ = 0.69
(Val/Val; Val/Met; Met/Met)			(*p* = 0.7)
**Handedness**	61; 3; 5	53; 5; 3	*χ*^2^ = 1.1
(Right; Left; Ambi)			(*p* = 0.58)

*Statistics indicate test of differences between sex groups*.

**Figure 1:**
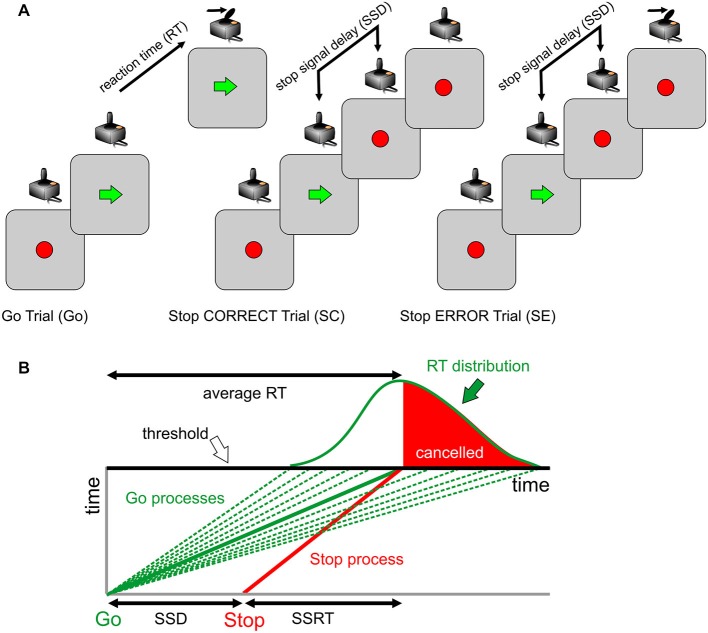
**Behavioral task: the race model. (A)** Trial types and sequence of visual stimuli presentation for each class. Only one direction of response (as indicated by the green arrow; Go signal) is presented. The red circle represents either the starting target or the Stop signal (when presented at the end of the stop signal delay (SSD)). **(B)** The race model explains the outcome of stop trials. Successful stopping is more probable for slower Go processes and less probable for faster Go processes. The family of green lines represents possible Go processes racing toward a fixed threshold, in different trials, with the Stop process indicated by the red line.

The behavioral setup and task used had the same structure of those previously described (Brunamonti et al., 2011, 2012, 2014). Before starting the session, each participant received written instructions and was made familiar with the apparatus and the task. Participants were seated in a dimly lit room with their eyes at a distance of about 45 cm from a PC monitor. Visual stimuli presentation and data acquisition were under the control of the freely available Psychtoolbox software (Matlab-based routines; www.psychtoolbox.org). A joystick was fixed to the table, aligned with subjects’ body midlines, and connected to a PC’s USB port. All trials started with the appearance of a visual cue (starting cue) in the middle of the screen (Figure 1A). The behavioral task consisted of a two-choice reaction time (RT) task with different trial types: in the more frequent trial type (Figure 1A; **Go trial**), participants moved, as quickly as possible, the joystick in the direction (either left or right) indicated by a visual stimulus (a spatially oriented arrow; go signal) presented after a variable holding time (800–1200 ms) and replacing the starting cue at the center of the display; in the less frequent trial (25% of total trials), an additional stimulus was presented (a red circle; stop signal) after a variable delay (stop signal delay; SSD) from the go signal and replacing it. Participants were told to abort the programmed movement upon the appearance of the stop signal on the display. If the subject correctly reacted to the stop signal, the trial was scored as “canceled” (Figure 1A; **Stop Correct Trial; SC**), whereas if the subject failed to abort the movement, the trial was scored as “not canceled” (Figure 1A; **Stop Error Trial; SE**). In Go trials, the time interval between the appearance of the go signal and the onset of movement corresponded to the RT. Successfully performed Go trials and Stop trials were marked by identical acoustic feedback. A different acoustic stimulus was exploited when the Go trial RT exceeded 500 ms as a warning to accomplish the primary task demand. Participants performed 3 blocks of 100 trials in a single session, with each block starting with a 50-ms SSD. Importantly, participants were told that because of the task design, it would not always be possible to withhold their response. During preliminary instructions, subjects were also made aware that the primary task consisted of responding as accurately as possible to the go signal, avoiding the interference of the stop signal with their performance.

### Behavioral Analyses

The race model (Logan, 1994) assumes that two processes race toward a fixed threshold during each stop trial: a variable go process initiated by the go signal appearance (Figure 1B, green lines) and a stop process initiated by the stop signal appearance (Figure 1B, red line). For a given stop process and a given SSD, there is a go process speed (Figure 1B, continuous red line) that corresponds to a 0.5 probability of success (i.e., 50% of stop trials canceled). When all Stop trials are considered, fast go processes correspond to an increased probability of Stop Error trials (Figure 1B, white region in the RT distribution), while slow go processes correspond to an increased probability of Stop Correct trials (Figure 1B, red region in the RT distribution).

After controlling for the validity of the assumptions of the race model in the data (Logan, 1994; see Results), a detailed performance analysis describes the probability of canceling the movement in stop trials. We used a staircase procedure to select the SSD in each Stop trial. The SSD was increased by 50 ms after each successfully canceled Stop trial and decreased by 50 ms after each non-canceled Stop trial. The procedure automatically adapts the SSD duration to the subject performance to obtain a probability of canceling a session that approximates 0.5 and is similar between subjects (for a similar approach, see Brunamonti et al., 2014).

We used the integrative method to estimate the length of time needed to cancel the planned movement—i.e., the stop signal RT (Figure 1B, SSRT)—because it is considered more robust (Verbruggen et al., 2013). The SSRT estimate is obtained by integrating the distribution of RTs in the Go trials until the integral equals the corresponding observed proportion of Stop Correct trials (probability of canceling) in the session. We also used the theoretical value of a 0.5 probability of canceling to obtain a different estimate (SSRT_0.5). However, using these SSRT_0.5 values, similar results have been obtained for the effects of both sex and genotype. Therefore, we decided not to include these results in the present report.

**Figure 2:**
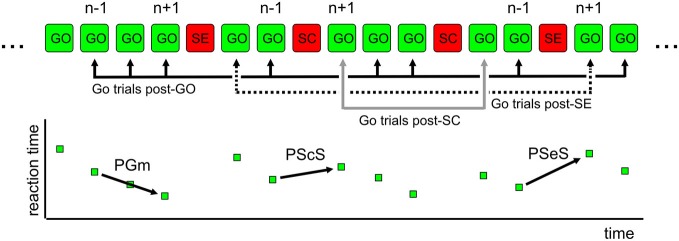
**Exemplificative sequence of trials-behavioral measures of proactive control**. In a countermanding task, trials display variable RTs. The elongation of REACTION TIME (RT) is higher in Go trials after a wrong stop trial (SE; PSeS) and smaller after a correct stop trial (SC; PScS). In a sequence of Go trials (GO-GO-GO), RT is expected to display a negative modulation (decreasing RT; PGm). In the top portion of the figure, typical triplets of trials used for the analysis are shown. For the computation of change in RT (Δt), the value in trial n − 1 is subtracted from the value in trial n + 1. For further details, see text.

We assessed, for each subject, the effect of the immediately preceding trial (**Go trial: GO; Stop Correct trial: SC; Stop Error trial: SE**) on the RT of Go trials (Figure 2). To exclude the contamination by global fluctuations in RT over the course of the test, we derived a difference between the trials immediately following and immediately preceding the critical trial using methods similar to those previously reported (Nelson et al., 2010; Dutilh et al., 2012). As an example, for SEs, we considered all GO-SE-GO triplets and subtracted trial n − 1 RT from trial n + 1 RT. The same procedure was adopted for GO and SC trials (GO-GO-GO and GO-SC-GO triplets, respectively). In this way, we were confident that the behavioral modulation related to the recent trial history and not to global fluctuations in RT was extracted. Indeed, a longer RT after a SC can be related to a general slowing down in a group of trials that allows one to correctly inhibit the response if a stop trial occurs and not to the stop trials *per se*. For each critical trial, we obtained a difference in RT (defined as Δt hereafter).

We defined **Post-Stop_error-slowing** (**PSeS**) as the amount of Δt for SE, **Post-Stop_correct-slowing** (**PScS**) as the amount of Δt for SC, and **Post-Go modulation (PGm)** as the amount of Δt for GO. Both PScS and PSeS represent a form of proactive control—i.e., the ability to adapt the response to the immediate context. They represent a measure of behavioral flexibility: after a change (a stop signal presentation) in the immediate history of the task, subjects may be more or less able to adjust their behavior in order to increase the probability of a correct performance, particularly if the adjustment is subsequent to an error trial. The successful strategy is to slow down the go process, moving the go process toward the family of green lines at the right of the average RT, corresponding to 0.5 probability of success (Figure 1B). Similarly, it is expected that a correct go trial will be followed by a shorter RT (a negative Δt) to optimize the speed-accuracy trade-off and reduce the time to obtain the correct trial.

### Genotyping

Genomic DNA was collected from buccal swabs and extracted using the Invitrogen PureLink Genomic DNA Mini kit (Invitrogen Life Technologies, Carlsbad, CA, USA; Cat # K1820-01) according to the manufacturer’s instructions. For privacy requirements, DNA samples were coded anonymously.

The SNP Val158Met of the COMT gene was genotyped using a TaqMan SNP Genotyping Assay (Applied Biosystems, CA, USA; Assay ID C_25746809_50, Lot Number 1607104). PCR amplification was carried out with 5–10 ng of genomic DNA, 1X TaqMan Genotyping master mix (Applied Biosystems), and allele probes labeled with 5′-VIC or 5′-FAM fluorophore. Amplification reaction conditions on an EcoTM Illumina thermal cycle were as follows: 1 cycle of 95°C for 10 min, followed by 50 cycles of 92°C for 15 s and 60°C for 1 min.

To confirm the COMT genotype, the Val158Met polymorphism was also assayed by the restriction fragment length polymorphism method. The polymorphic region was amplified by polymerase chain reaction (PCR) using the following primers: COMT_For 5′ GGGGCCTACTGTGGCTACTC 3′ and COMT_Rev 5′ TTTTTCCAGGTCTGACAACG 3′. The Val and Met alleles were discriminated by digesting the PCR product with NlaIII restriction enzyme, followed by 2% agarose gel electrophoresis and visualization by ethidium bromide staining. The undigested PCR product (109 bp) carried the G variant (Val), while the digested product, giving two fragments of 96 and 13 bp, respectively, contained the A allele (Met).

Several replicates, reference DNA samples, and negative controls without DNA were included to ensure the accuracy of the SNP genotypes.

## Results

As a first step, we controlled behavioral performance in the different groups of subjects to assess the independence of the go and stop processes (Logan, 1994). All subjects displayed a shorter mean RT of Stop Error trials (SE) than the mean RT of Go trials (GO). This result was statistically validated both at the population level (SE (mean ± s.d.): 373.04 ± 22.7; GO (mean ± s.d.): 418.9 ± 26.9; Kolmogorov-Smirnov test, *p* < 0.001) and for 111/130 subjects (85.4%; Kolmogorov-Smirnov test, *p* < 0.05).

No significant differences were observed in the proportion of participants violating the independence assumption of the race model when sorted by genotype and sex (Pearson *χ*^2^ test; *p* > 0.05). We also demonstrated at the population level an increased duration of RT in stop error trials with SSD (one-way ANOVA *F*_(5,3991)_ = 71.65, *p* < 0.0001). A regression analysis showed a significant trend (*p* < 0.05) emerging for 50/130 subjects (38.5%) when considering SSD, with at least 6 trials presented. Finally, we tested the validity of the model by looking for differences in the observed values of RT in SEs from values predicted by the model at each SSD. We observed a significant difference in 49/130 subjects (37.7%) (Kolmogorov-Smirnov test, *p* < 0.05). As suggested by simulation studies (Band et al., 2003), a mismatch between an observed RT and predicted RT in SEs can not be considered a valid test for violation of the independence assumption. Therefore, all subjects were included in the database for the successive analyses.

For each variable in Table 2, we performed separate two-way analyses of covariance (ANCOVAs), having genotype (Val-Val, Val-Met, Met-Met) and sex (M, F) as between-subject factors and age as a covariate, finding no significant effects. Thus, none of the parameters examined that were related to the reactive control were influenced by either the sex or the genotype.

**Table 2:** **Descriptive statistics of the behavioral performance for each experimental group**.

	Val/Val		Val/Met		Met/Met	
	Male	Female	Male	Female	Male	Female
**SSRT** Mean (s.e.)	262 (8.9)	278.1 (10.1)	260.9 (6.4)	268.8 (7.0)	268.1 (9.5)	259 (8.9)
**RT-Go** Mean (s.e.)	411.2 (6.3)	429.1 (7.2)	417.7 (4.5)	422.1 (5.0)	419.7 (6.7)	416.5 (6.3)
**P (canceled)** Mean (s.e.)	0.55 (0.01)	0.55 (0.01)	0.53 (0.01)	0.55 (0.01)	0.53 (0.01)	0.53 (0.01)
**SSD** Mean (s.e.)	132.4 (8.8)	127.9 (1.0)	141.9 (6.3)	133.6 (6.9)	137.2 (9.3)	139.5 (8.8)
**RT-SE** Mean (s.e.)	365.7 (5.4)	379.5 (6.1)	372 (3.8)	376.4 (4.2)	373.1 (5.9)	371.8 (5.4)

*RT-Go indicates reaction time in all Go trials. RT-SE indicates reaction time in stop error trials*.

[RT in Go trials: Factor Sex *F*_(1,122)_ = 2.28, *p* = 0.13; Factor Genotype *F*_(2,122)_ = 0.08, *p* = 0.95; Sex * Genotype *F*_(2,122)_ = 1.35, *p* = 0.26. Probability of canceling in a stop trial: Factor Sex *F*_(1,122)_ = 0.6, *p* = 0.45; Factor Genotype *F*_(2,122)_ = 1.1, *p* = 0.34; Sex * Genotype *F*_(2,122)_ = 0.95, *p* = 0.38. For the average SSD: Factor Sex *F*_(1,122)_ = 0.001, *p* = 0.97; Factor Genotype *F*_(2,122)_ = 0.2, *p* = 0.82; Sex * Genotype *F*_(2,122)_ = 0.27, *p* = 0.75. For the SSRT, Factor Sex *F*_(1,122)_ = 0.15, *p* = 0.7; Factor Genotype *F*_(2,122)_ = 0.09, *p* = 0.91; Sex * Genotype *F*_(2,122)_ = 1.04, *p* = 0.35. For the RT-SE, Factor Sex *F*_(1,121)_ = 2.8, *p* = 0.09; Factor Genotype *F*_(2,121)_ = 0.08, *p* = 0.92; Sex * Genotype *F*_(2,121)_ = 1.13, *p* = 0.32].

**Figure 3:**
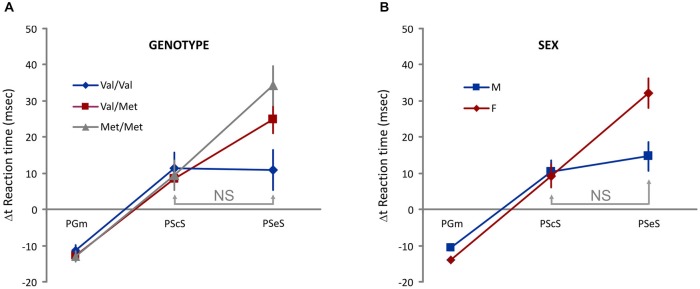
**Evidence of genotype- and sex-dependent difference in the proactive control. (A)** An increasing Δt is observed for Met/Met and Val/Met subjects. Val/Val subjects do not display differences between PScS and PSeS. **(B)** An increasing Δt is observed for female subjects. Male subjects do not display differences between PScS and PSeS; NS = no significant difference [Bonferroni *post hoc* tests. Interaction Type of Δt * Genotype: Val/Val: PScS vs. PSeS *p* = 1; PScS vs. PGm and PSeS vs. PGm ps < 0.05; Val/Met: PScS vs. PSeS; PScS vs. PGm and PSeS vs. PGm ps < 0.05; Met/Met: PScS vs. PSeS; PScS vs. PGm and PSeS vs. PGm ps < 0.005. Interaction Type of Δt * Sex: Males: PScS vs. PSeS *p* = 1; PScS vs. PGm and PSeS vs. PGm ps < 0.00005; Female: PScS vs. PSeS; PScS vs. PGm and PSeS vs. PGm *ps* < 0.00005].

To examine the proactive control of movement, we considered the between-trial change (Δt) in Go trials RT as a dependent variable, two between-subject factors [Genotype (Val-Val, Val-Met, Met-Met) and Sex (M, F)], one within-subject factor (different types of Δt: **PGm, PScS**, and **PSeS**; Figure 2), and one covariate (Age). We found no significant main effects of Sex, Genotype, and Type of Δt. We found significant interactions: Sex * Type of Δt (*F*_(2,242)_ = 4.8, *p* = 0.008) and Genotype * Type of Δt (*F*_(4,242)_ = 2.8, *p* = 0.03). Additional analysis (Bonferroni *post hoc* tests) showed that (see Figure 3 for details) when groups were sorted for genotype (Figure 3A), both Met/Met and Val/Met subjects displayed a negative Δt for PGm and a progressively increasing positive Δt for PScS and PSeS. Val/Val subjects, instead, were those with no significant differences between PScS and PSeS. A similar feature emerged when subjects were sorted for sex (Figure 3B): male subjects did not slow down after an error trial compared to a stop correct trial, whereas an increasing trend was observed for females.

Overall, these results show that the presence of a stop error trial in the recent task history is less effective in Val/Val subjects and in male subjects.

## Discussion

Exploiting a classic version of the countermanding task, validated in different studies by our group (Brunamonti et al., 2011, 2012, 2014), in this study, we demonstrate that: (i) sex-dependent and COMT genotype-dependent differences are detected for proactive control but not for reactive control in the countermanding task; and (ii) the amount of post-error slowing in the countermanding task shows sex-dependent and genotype-dependent differences. More precisely, we report the existence of an important difference in the cognitive control of motor responses between males and females and in Val/Val subjects: both males and Val/Val homozygotes showed a lower ability to adapt the response speed after an error trial. In our data, no interaction emerged between genotype and sex for any of the measures.

We did not observe any effect of either sex or the COMT Val158Met SNP on the duration of SSRT, confirming what was previously reported by Congdon and Canli (2005) and White et al. (2014). On the other hand, an advantage of Val allele carriers for SSRT duration has been previously reported in a modified version of the countermanding paradigm (the flanker-stop-signal paradigm; Krämer et al., 2007). However, compared to our study, the number of total subjects in Kramer’s study was low (*n* = 40), and most importantly, participants were not matched for sex (6/14 males in the Val/Val group; 14/26 males in the Met/Met group). In the recent study of White et al. (2014), a difference in BOLD activation of key frontal areas (inferior frontal cortex, supplementary motor area, and pre-supplementary motor area) was observed after sorting adolescent subjects tested in the countermanding task by sex and COMT genotype. Even without significant behavioral differences observed, male Val/Val subjects were those with the strongest BOLD activation. In the study of White et al. (2014), proactive control was not analyzed.

Overall, the difference observed for reactive and proactive inhibition suggests either that the two processes are influenced differently by the function of dopaminergic systems or that they are under the control of different neural systems (Chen et al., 2010; Schall and Godlove, 2012; Stuphorn and Emeric, 2012; Marcos et al., 2013; Pani et al., 2014). In the latter case, it is tempting to hypothesize that the influence of dopamine activity in these neural systems might be different.

In our study, Val/Val homozygotes and male subjects display less pronounced post-error slowing (PSeS), whereas Met-carriers (Val/Met and Met/Met) and female subjects display larger slowing. Slowing the response after an error is believed to be a direct measure of cognitive control (Rabbitt, 1966) and is observed in a wide range of tasks, including the Simon task (King et al., 2010; Danielmeier et al., 2011), the flanker task (Debener et al., 2005; Krämer et al., 2007), the Stroop task (Gehring and Fencsik, 2001), and the stop task (Mirabella et al., 2006; Emeric et al., 1997; Brunamonti et al., 2014). Since the extent of slowing also depends on the number of errors in the block (Steinborn et al., 2012), to avoid possible interference of the overall performance in the stop task, we used a staircase algorithm that dynamically adapts SSD selections to the probability of error (Band et al., 2003), ensuring a probability of about 0.5 for each subject.

According to the dual mechanisms of control (DMC) model (Braver et al., 2007; Braver, 2012), proactive control is dependent on normal prefrontal cortex function and on dopamine availability, reflecting active maintenance of task-relevant information after the completion of each trial. Thus, in proactive control, the advantage of Met allele carriers that we observed was expected and confirmed a recent report (Jaspar et al., 2014). Additionally, our data pinpoint to a “female advantage” that deserves attention.

At first blush, our results seem to be inconsistent with the evidence that the post-error slowing is not influenced by both dopamine agonists, such as D-amphetamine (de Bruijn et al., 2004), and dopamine antagonists, such as haloperidol (de Bruijn et al., 2006). Re-interpreting these findings in light of our data, it is tempting to hypothesize that dopaminergic activity (and COMT efficiency) could be dependent on sex-associated differences, perhaps able to influence the background level of dopamine. In fact, the variable “sex” was not controlled in these studies. Unfortunately, our data are not definitive in this respect, and the lack of an interaction between sex and genotype will require further studies including a larger number of subjects sorted by both COMT genotype and sex.

The prefrontal cortex is the region of the brain that is most influenced by the level of activity of COMT and dopamine concentration (Matsumoto et al., 2003; Tunbridge et al., 2004). For the relationship between dopamine level and prefrontal function, an inverted U-shape model has been proposed (Arnsten, 1997; Williams and Castner, 2006; Mier et al., 2010). This model assumes that normal prefrontal cortex functions rely on an optimal range of dopamine acting on the D1 class of dopamine receptors. Conversely, “too little” or “too much” D1 stimulation has detrimental effects on functions that are under the control of the prefrontal cortex and is typical of subjects falling in the two opposite sides of the U-curve (considered to be under the influence of the D2 class of receptors), respectively. The worse prefrontal cortex functioning of these subjects is associated with better functioning at the sub-cortical level. Anatomical and neurochemical sex-dependent differences influence the background level of dopamine, likely changing the effect of COMT on the U-shape curve (Jacobs and D’Esposito, 2011). In this regard, a recent study describes how COMT and sex strongly interact to determine the thickness of the prefrontal cortex in both humans and rodents (Sannino et al., 2014).

Sex dimorphic activity of COMT was originally explained by the finding that 17-β-estradiol administration decreased COMT activity (Cohn and Axelrod, 1971; Jiang et al., 2003). Thus, estrogens have the same effect on COMT as the Met allele. On the other hand, COMT catabolizes catechol group-containing estrogens (Creveling, 2003), pinpointing a reciprocal genotype-influenced interaction between COMT activity and estrogens. However, several studies showed that a sex-dependent difference in COMT transcript and protein levels is absent in the brain, leading to the conclusion that this interaction may not apply to the brain, as it does in peripheral tissue (Chen et al., 2004; Tunbridge et al., 2004; Dempster et al., 2006). Several hypotheses are currently under investigation to explain the sex dimorphic activity of COMT in the brain. As an example, the level of S-adenosylmethionine (SAM), the donor of the methyl group that is required for COMT enzymatic activity (Zhu, 2002), may differ between males and females, as indicated by the finding that SAM supplementation has a beneficial effect on depressive symptoms in females but not in males (Strous et al., 2009). Moreover, several sex-specific differences in biochemical pathways of the dopaminergic system have been reported (Kaasinen et al., 2001; Mozley et al., 2001; Laakso et al., 2002; Munro et al., 2006). For example, females have a significantly greater proportion (about 50%) of dopaminergic cells in the mesocortical dopaminergic pathway projecting to the prefrontal cortex (Kritzer and Creutz, 2008). These data suggest a higher tonic level of dopamine in female subjects.

The combined effect of sex and the COMT Val158/Met SNP in influencing human behavior is in agreement with recent observations on prepulse inhibition (PPI) of the startle reflex. This represents an additional measure of inhibitory control used in both healthy subjects and patients (Calkins et al., 2007) that is known to be regulated by dopaminergic transmission (Geyer et al., 2002). In a study in which only males were recruited, a clear dependence of PPI on dopamine transmission in prefrontal cortex has emerged: Val/Val (male) subjects displayed a significantly reduced PPI (Roussos et al., 2008), paralleling our main result. A second study of the same group (Giakoumaki et al., 2008) reported that tolcapone (a COMT suppressor) caused an increase of PPI in Val/Val male subjects and found a tendency of the opposite effect in Met/Met subjects. Conversely, no effect of COMT polymorphism on PPI duration has been reported by Montag et al. (2008) in a group formed by only female subjects.

In summary, our data suggest that sex-dependent differences in proactive control of response inhibition could emerge for the Val/Val COMT Val158/Met SNP. Our results emphasize the importance of matching experimental groups for sex when studying the interaction between sex and genetic variations. Also, our findings could be relevant for understanding the potential protective effect of estrogens on pathologies where the dopamine level is crucial, such as schizophrenia.

## Conflict of Interest Statement

The authors declare that the research was conducted in the absence of any commercial or financial relationships that could be construed as a potential conflict of interest.

## References

[B1] Arnsten A. F. (1997). Catecholamine regulation of the prefrontal cortex. J. Psychopharmacol. 11, 151–162. 10.1177/026988119701100208 9208378

[B2] Aron A. R. (2011). From reactive to proactive and selective control: developing a richer model for stopping inappropriate responses. Biol. Psychiatry 69, e55–e68. 10.1016/j.biopsych.2010.07.024 20932513 PMC3039712

[B3] Aron A. R. Poldrack R. A. (2006). Cortical and subcortical contributions to stop signal response inhibition: role of the subthalamic nucleus. J. Neurosci. 26, 2424–2433. 10.1523/jneurosci.4682-05.2006 16510720 PMC6793670

[B4] Band G. P. van der Molen M. W. Logan G. D. (2003). Horse-race model simulations of the stop-signal procedure. Acta Psychol. (Amst) 112, 105–142. 10.1016/s0001-6918(02)00079-3 12521663

[B5] Battaglia-Mayer A. Buiatti T. Caminiti R. Ferraina S. Lacquaniti F. Shallice T. (2014). Correction and suppression of reaching movements in the cerebral cortex: physiological and neuropsychological aspects. Neurosci. Biobehav. Rev. 42, 232–251. 10.1016/j.neubiorev.2014.03.002 24631852

[B6] Braver T. S. (2012). The variable nature of cognitive control: a dual-mechanisms framework. Trends Cogn. Sci. 16, 106–113. 10.1016/j.tics.2011.12.010 22245618 PMC3289517

[B7] Braver T. S. Gray J. R. Burgess G. C. (2007). “Explaining the many varieties of working memory variation: dual mechanisms of cognitive control,” in Variation in Working Memory, eds Conway A. R. A. Jarrold C. Kane M. J. Miyake A. Towse J. N. (Oxford: Oxford University Press), 76–106.

[B8] Brunamonti E. Chiricozzi F. R. Clausi S. Olivito G. Giusti M. A. Molinari M.. (2014). Cerebellar damage impairs executive control and monitoring of movement generation. PLoS One 9:e85997. 10.1371/journal.pone.0085997 24465830 PMC3895022

[B9] Brunamonti E. Ferraina S. Paré M. (2012). Controlled movement processing: evidence for a common inhibitory control of finger, wrist and arm movements. Neuroscience 215, 69–78. 10.1016/j.neuroscience.2012.04.051 22554783

[B10] Brunamonti E. Pani P. Papazachariadis O. Onorati P. Albertini G. Ferraina S. (2011). Cognitive control of movement in down syndrome. Rev. Dev. Disabil. 32, 1792–1797. 10.1016/j.ridd.2011.03.008 21482066

[B11] Calkins M. E. Dobie D. J. Cadenhead K. S. Olincy A. Freedman R. Green M. F.. (2007). The consortium on the genetics of endophenotypes in schizophrenia: model recruitment, assessment and endophenotyping methods for a multisite collaboration. Schizophr. Bull. 33, 33–48. 10.1093/schbul/sbl044 17035358 PMC2632302

[B12] Chambers C. D. Garavan H. Bellgrove M. A. (2009). Insights into the neural basis of response inhibition from cognitive and clinical neuroscience. Neurosci. Biobehav. Rev. 33, 631–646. 10.1016/j.neubiorev.2008.08.016 18835296

[B13] Chen J. S. Lipska B. K. Halim N. Ma Q. D. Matsumoto M. Melhem S.. (2004). Functional analysis of genetic variation in catechol-O-methyltransferase (COMT): effects on mRNA, protein and enzyme activity in postmortem human brain. Am. J. Hum. Genet. 75, 807–821. 10.1086/425589 15457404 PMC1182110

[B14] Chen X. Scangos K. W. Stuphorn V. (2010). Supplementary motor area exerts proactive and reactive control of arm movements. J. Neurosci. 30, 14657–14675. 10.1523/JNEUROSCI.2669-10.2010 21048123 PMC2990193

[B15] Cohn C. K. Axelrod J. (1971). The effect of estradiol on catechol-O-methyltransferase activity in rat liver. Life Sci. 10, 1351–1354. 10.1016/0024-3205(71)90335-3 5144295

[B16] Congdon E. Canli T. (2005). The endophenotype of impulsivity: reaching consilience through behavioral, genetic and neuroimaging approaches. Behav. Cogn. Neurosci. Rev. 4, 262–281. 10.1177/1534582305285980 16585800

[B17] Creveling C. R. (2003). The role of catechol-O-methyltransferase in the inactivation of catecholestrogen. Cell. Mol. Neurobiol. 23, 289–291. 10.1023/A:1023680302975 12825827 PMC11530145

[B18] Danielmeier C. Eichele T. Forstmann B. U. Tittgemeyer M. Ullsperger M. (2011). Posterior medial frontal cortex activity predicts post-error adaptations in task-related visual and motor areas. J. Neurosci. 31, 1780–1789. 10.1523/JNEUROSCI.4299-10.2011 21289188 PMC6623722

[B19] Debener S. Ullsperger M. Siegel M. Fiehler K. von Cramon D. Y. Engel A. K. (2005). Trial-by-trial coupling of concurrent electroencephalogram and functional magnetic resonance imaging identifies the dynamics of performance monitoring. J. Neurosci. 25, 11730–11737. 10.1523/jneurosci.3286-05.2005 16354931 PMC6726024

[B20] de Bruijn E. R. Hulstijn W. Verkes R. J. Ruigt G. S. Sabbe B. G. (2004). Drug-induced stimulation and suppression of action monitoring in healthy volunteers. Psychopharmacology 177, 151–160. 10.1007/s00213-004-1915-6 15578258

[B21] de Bruijn E. R. Sabbe B. G. Hulstijn W. Ruigt G. S. Verkes R. J. (2006). Effects of antipsychotic and antidepressant drugs on action monitoring in healthy volunteers. Brain Res. 1105, 122–129. 10.1016/j.brainres.2006.01.006 16499887

[B22] Dempster E. L. Mill J. Craig I. W. Collier D. A. (2006). The quantification of COMT mRNA in post mortem cerebellum tissue: diagnosis, genotype, methylation and expression. BMC Med. Genet. 7:10. 10.1186/1471-2350-7-10 16483362 PMC1456954

[B23] Dickinson D. Elvevåg B. (2009). Genes, cognition and brain through a comt lens. Neuroscience 164, 72–87. 10.1016/j.neuroscience.2009.05.014 19446012 PMC2760675

[B24] Dutilh G. van Ravenzwaaij D. Nieuwenhuis S. van der Maas H. L. J. Forstmann B. U. Wagenmakers E. J. (2012). How to measure post-error slowing: a confound and a simple solution. J. Math. Psychol. 56, 208–216. 10.1016/j.jmp.2012.04.001

[B26] Eme R. F. (2007). Sex differences in child-onset, life-course-persistent conduct disorder. A review of biological influences. Clin. Psychol. Rev. 27, 607–627. 10.1016/j.cpr.2007.02.001 17331630

[B27] Emeric E. E. Brown J. W. Boucher L. Carpenter R. H. S. Hanes D. P. Harris R.. (1997). Influence of history on saccade countermanding performance in humans and macaque monkeys. Vision Res. 47, 35–49. 17081584 10.1016/j.visres.2006.08.03 PMC1815391

[B28] Garavan H. Hester R. Murphy K. Fassbender C. Kelly C. (2006). Individual differences in the functional neuroanatomy of inhibitory control. Brain Res. 1105, 130–142. 10.1016/j.brainres.2006.03.029 16650836

[B29] Garavan H. Ross T. J. Murphy K. Roche R. A. Stein E. A. (2002). Dissociable executive functions in the dynamic control of behavior: inhibition, error detection and correction. Neuroimage 17, 1820–1829. 10.1006/nimg.2002.1326 12498755

[B30] Gehring W. J. Fencsik D. E. (2001). Functions of the medial frontal cortex in the processing of conflict and errors. J. Neurosci. 21, 9430–9437. 11717376 10.1523/JNEUROSCI.21-23-09430.2001 PMC6763895

[B31] Geyer M. A. McIlwain K. L. Paylor R. (2002). Mouse genetic models for prepulse inhibition: an early review. Mol. Psychiatry 7, 1039–1053. 10.1038/sj.mp.4001159 12476318

[B32] Giakoumaki S. G. Roussos P. Bitsios P. (2008). Improvement of prepulse inhibition and executive function by the COMT inhibitor tolcapone depends on COMT Val158Met polymorphism. Neuropsychopharmacology 33, 3058–3068. 10.1038/npp.2008.82 18536698

[B33] Gogos J. A. Morgan M. Luine V. Santha M. Ogawa S. Pfaff D.. (1998). Catechol-O-methyltransferase-deficient mice exhibit sexually dimorphic changes in catecholamine levels and behavior. Proc. Natl. Acad. Sci. U S A 95, 9991–9996. 10.1073/pnas.95.17.9991 9707588 PMC21449

[B34] Harrison P. J. Tunbridge E. M. (2008). Catechol-O-Methyltransferase (COMT): a gene contributing to sex differences in brain function and to sexual Dimorphism in the predisposition to psychiatric disorders. Neuropsychopharmacology 33, 3037–3045. 10.1038/sj.npp.1301543 17805313

[B35] Hart H. Radua J. Nakao T. Mataix-Cols D. Rubia K. (2013). Meta-analysis of functional magnetic resonance imaging studies of inhibition and attention in attention-deficit/hyperactivity disorder: exploring task-specific, stimulant medication and age effects. JAMA Psychiatry 70, 185–198. 10.1001/jamapsychiatry.2013.277 23247506

[B36] Jacobs E. D’Esposito M. (2011). Estrogen shapes dopamine-dependent cognitive processes: implications for women’s health. J. Neurosci. 31, 5286–5293. 10.1523/JNEUROSCI.6394-10.2011 21471363 PMC3089976

[B37] Jaspar M. Genon S. Muto V. Meyer C. Manard M. Dideberg V.. (2014). Modulating effect of COMT genotype on the brain regions underlying proactive control process during inhibition. Cortex 50, 148–161. 10.1016/j.cortex.2013.06.003 23859480

[B38] Jiang H. Xie T. Ramsden D. B. Ho S. L. (2003). Human catechol-O-methyltransferase down-regulation by estradiol. Neuropharmacology 45, 1011–1018. 10.1016/s0028-3908(03)00286-7 14573393

[B39] Kaasinen V. Någren K. Hietala J. Farde L. Rinne J. O. (2001). Sex differences in extrastriatal dopamine D2-like receptors in the human brain. Am. J. Psychiatry 158, 308–311. 10.1176/appi.ajp.158.2.308 11156817

[B40] Karayiorgou M. Sobin C. Blundell M. L. Galke B. L. Malinova L. Goldberg P.. (1999). Family-based association studies support a sexually dimorphic effect of COMT and MAOA on genetic susceptibility to obsessive-compulsive disorder. Biol. Psychiatry 45, 1178–1189. 10.1016/s0006-3223(98)00319-9 10331110

[B41] Kessler R. C. Angermeyer M. Anthony J. C. DE Graaf R. Demyttenaere K. Gasquet I.. (2007). Lifetime prevalence and age-of-onset distributions of mental disorders in the World Health Organization’s World Mental Health Survey Initiative. World Psychiatry 6, 168–176. 18188442 PMC2174588

[B42] King J. A. Korb F. M. von Cramon D. Y. Ullsperger M. (2010). Post-error behavioral adjustments are facilitated by activation and suppression of task-relevant and task-irrelevant information processing. J. Neurosci. 30, 12759–12769. 10.1523/JNEUROSCI.3274-10.2010 20861380 PMC6633589

[B43] Krämer U. M. Cunillera T. Càmara E. Marco-Pallarés J. Cucurell D. Nager W.. (2007). The impact of catechol-O-methyltransferase and dopamine D4 receptor genotypes on neurophysiological markers of performance monitoring. J. Neurosci. 27, 14190–14198. 10.1523/jneurosci.4229-07.2007 18094258 PMC6673506

[B44] Kritzer M. F. Creutz L. M. (2008). Region and sex differences in constituent dopamine neurons and immunoreactivity for intracellular estrogen and androgen receptors in mesocortical projections in rats. J. Neurosci. 28, 9525–9535. 10.1523/JNEUROSCI.2637-08.2008 18799684 PMC2613180

[B45] Laakso A. Vilkman H. Bergman J. Haaparanta M. Solin O. Syvälahti E.. (2002). Sex differences in striatal presynaptic dopamine synthesis capacity in healthy subjects. Biol. Psychiatry 52, 759–763. 10.1016/s0006-3223(02)01369-0 12372667

[B46] Li C. S. Huang C. Constable R. T. Sinha R. (2006). Gender differences in the neural correlates of response inhibition during a stop signal task. Neuroimage 32, 1918–1929. 10.1016/j.neuroimage.2006.05.017 16806976

[B47] Li C. S. Zhang S. Duann J. R. Yan P. Sinha R. Mazure C. M. (2009). Gender differences in cognitive control: an extended investigation of the stop signal task. Brain Imaging Behav. 3, 262–276. 10.1007/s11682-009-9068-1 19701485 PMC2728908

[B48] Logan G. D. (1994). “On the ability to inhibit thought and action: a users’ guide to the stop signal paradigm,” in Inhibitory Processes in Attention, Memory and Language, eds Dagenbach D. Carr T. H. (San Diego: Academic Press), 189–239.

[B49] Marcos E. Pani P. Brunamonti E. Deco G. Ferraina S. Verschure P. (2013). Neural variability in premotor cortex is modulated by trial history and predicts behavioral performance. Neuron 78, 249–255. 10.1016/j.neuron.2013.02.006 23622062

[B50] Matsumoto M. Weickert C. S. Akil M. Lipsa B. K. Hyde T. M. Herman M. M.. (2003). Catechol O-methyltransferase mRNA expression in human and rat brain: evidence for a role in cortical neuronal function. Neuroscience 116, 127–137. 10.1016/s0306-4522(02)00556-0 12535946

[B51] Meyer-Lindenberg A. Nichols T. Callicott J. H. Ding J. Kolachana B. Buckholtz J.. (2006). Impact of complex genetic variation in COMT on human brain function. Mol. Psychiatry 11, 867–877. 10.1038/sj.mp.4001860 16786032

[B52] Mier D. Kirsch P. Meyer-Lindberg A. (2010). Neural substrates of pleiotropic action of genetic variation in COMT: a meta-analysis. Mol. Psychiatry 15, 918–927. 10.1038/mp.2009.36 19417742

[B53] Mirabella G. Pani P. Paré M. Ferraina S. (2006). Inhibitory control of reaching movements in humans. Exp. Brain Res. 174, 240–255. 10.1007/s00221-006-0456-0 16636792

[B54] Montag C. Buckholtz J. W. Hartmann P. Merz M. Burk C. Hennig J.. (2008). COMT genetic variation affects fear processing: psychophysiological evidence. Behav. Neurosci. 122, 901–909. 10.1037/0735-7044.122.4.901 18729643

[B55] Mozley L. H. Gur R. C. Mozley P. D. Gur R. E. (2001). Striatal dopamine transporters and cognitive functioning in healthy men and women. Am. J. Psychiatry 158, 1492–1499. 10.1176/appi.ajp.158.9.1492 11532737

[B56] Munro C. A. McCaul M. E. Wong D. F. Oswald L. M. Zhou Y. Brasic J.. (2006). Sex differences in striatal dopamine release in healthy adults. Biol. Psychiatry 59, 966–974. 10.1016/j.biopsych.2006.01.008 16616726

[B57] Nelson M. J. Boucher L. Logan G. D. Palmeri T. J. Schall J. D. (2010). Nonindependent and nonstationary response times in stopping and stepping saccade tasks. Atten. Percept. Psychophys. 72, 1913–1929. 10.3758/APP.72.7.1913 20952788 PMC3237060

[B58] Newman J. P. MacCoon D. G. Vaughn L. J. Sadeh N. (2005). Validating a distinction between primary and secondary psychopathy with measures of Gray’s BIS and BAS constructs. J. Abnorm. Psychol. 114, 319–323. 10.1037/0021-843x.114.2.319 15869363

[B59] Oldfield R. C. (1971). The assessment and analysis of handedness: the Edinburgh inventory. Neuropsychologia 9, 97–113. 10.1016/0028-3932(71)90067-4 5146491

[B60] Pani P. Di Bello F. Brunamonti E. D’Andrea V. Papazachariadis O. Ferraina S. (2014). Alpha- and beta-band oscillations subserve different processes in reactive control of limb movements. Front. Behav. Neurosci. 8:383. 10.3389/fnbeh.2014.00383 25414649 PMC4220745

[B61] Rabbitt P. M. (1966). Error and error correction in choice-response tasks. J. Exp. Psychol. 71, 264–272. 10.1037/h0022853 5948188

[B62] Rabbitt P. M. Phillips S. (1967). Error-detection and correction latencies as a function of S-R compatibility. Q. J. Exp. Psychol. 19, 37–42. 10.1080/14640746708400065 6041680

[B63] Rieger M. Gauggel S. Burmeister K. (2003). Inhibition of ongoing responses following frontal, nonfrontal and basal ganglia lesions. Neuropsychology 17, 272–282. 10.1037/0894-4105.17.2.272 12803433

[B64] Robbins T. W. Gillan C. M. Smith D. G. de Wit S. Ersche K. D. (2012). Neurocognitive endophenotypes of impulsivity and compulsivity: towards dimensional psychiatry. Trends Cogn. Sci. 16, 81–91. 10.1016/j.tics.2011.11.009 22155014

[B65] Roussos P. Giakoumaki S. G. Rogdaki M. Pavlakis S. Frangou S. Bitsios P. (2008). Prepulse inhibition of the startle reflex depends on the catechol O-methyltransferase Val158Met gene polymorphism. Psychol. Med. 38, 1651–1658. 10.1017/s0033291708002912 18261249

[B66] Rubia K. Halari R. Cubillo A. Smith A. B. Mohammad A. M. Brammer M.. (2011). Methylphenidate normalizes fronto-striatal underactivation during interference inhibition in medication-naive boys with attention-deficit hyperactivity disorder. Neuropsychopharmacology 36, 1575–1586. 10.1038/npp.2011.30 21451498 PMC3116801

[B67] Sannino S. Gozzi A. Cerasa A. Piras F. Scheggia D. Managò F.. (2014). COMT genetic reduction produces sexually divergent effects on cortical anatomy and working memory in mice and humans. Cereb. Cortex [Epub ahead of print]. 10.1093/cercor/bhu053 24658585 PMC4542698

[B68] Schall J. D. Godlove D. C. (2012). Current advances and pressing problems in studies of stopping. Curr. Opin. Neurobiol. 22, 1012–1021. 10.1016/j.conb.2012.06.002 22749788 PMC3496825

[B69] Steinborn M. B. Flehmig H. C. Bratzke D. Schröter H. (2012). Error reactivity in self-paced performance: highly-accurate individuals exhibit largest post-error slowing. Q. J. Exp. Psychol. (Hove) 4, 624–631. 10.1080/17470218.2012.660962 22463389

[B70] Strous R. D. Ritsner M. S. Adler S. Ratner Y. Maayan R. Kotler M.. (2009). Improvement of aggressive behavior and quality of life impairment following S-adenosyl-methionine (SAM-e) augmentation in schizophrenia. Eur. Neuropsychopharmacol. 19, 14–22. 10.1016/j.euroneuro.2008.08.004 18824331

[B71] Stuphorn V. (2015). Neural mechanisms of response inhibition. Curr. Opin. Behav. Sci. 1, 64–71. 10.1016/j.cobeha.2014.10.009

[B72] Stuphorn V. Emeric E. E. (2012). Proactive and reactive control by the medial frontal cortex. Front. Neuroeng. 6:9. 10.3389/fneng.2012.00009 22723779 PMC3378012

[B73] Thakkar K. N. Congdon E. Poldrack R. A. Sabb F. W. London E. D. Cannon T. D.. (2014). Women are more sensitive than men to prior trial events on the stop-signal task. Br. J. Psychol. 105, 254–272. 10.1111/bjop.12034 24754812 PMC4000536

[B74] Tunbridge E. M. Bannerman D. M. Sharp T. Harrison P. J. (2004). Catechol-o-methyltransferase inhibition improves set-shifting performance and elevates stimulated dopamine release in the rat prefrontal cortex. J. Neurosci. 24, 5331–5335. 10.1523/jneurosci.1124-04.2004 15190105 PMC6729311

[B75] Verbruggen F. Chambers C. D. Logan G. D. (2013). Fictitious inhibitory differences: how skewness and slowing distort the estimation of stopping latencies. Psychol. Sci. 24, 352–362. 10.1177/0956797612457390 23399493 PMC3724271

[B76] Verbruggen F. Logan G. D. (2009). Proactive adjustments of response strategies in the stop-signal paradigm. J. Exp. Psychol. Hum. Percept. Perform. 35, 835–854. 10.1037/a0012726 19485695 PMC2690716

[B77] White T. P. Loth E. Rubia K. Krabbendam L. Whelan R. Banaschewski T.. (2014). Sex differences in COMT polymorphism effects on prefrontal inhibitory control in adolescence. Neuropsychopharmacology 39, 2560–2569. 10.1038/npp.2014.107 24820538 PMC4207335

[B78] Williams G. V. Castner S. A. (2006). Under the curve: critical issues for elucidating D1 receptor functions in working memory. Neuroscience 139, 263–276. 10.1016/j.neuroscience.2005.09.028 16310964

[B79] Zhu B. T. (2002). Catechol-O-Methyltransferase (COMT)-mediated methylation metabolism of endogenous bioactive catechols and modulation by endobiotics and xenobiotics: importance in pathophysiology and pathogenesis. Curr. Drug Metab. 3, 321–349. 10.2174/1389200023337586 12083324

